# Supplementary material for: Evaluation of subretinally delivered Cas9 ribonucleoproteins in murine and porcine animal models highlights key considerations for therapeutic translation of genetic medicines
Source: bioRxiv. 2024 Dec 30:2024.12.30.630799. Preprint. [Version 1] doi: 10.1101/2024.12.30.630799 (PMC11722268; doi:10.1101/2024.12.30.630799)
Supplement: 1 [file NIHPP2024.12.30.630799v1-supplement-1.pdf]

bioRxiv preprint doi: https://doi.org/10.1101/2024.12.30.630799; this version posted December 31, 2024. The copyright holder for this preprint (which was not certified by peer review) is the author/funder, who has granted bioRxiv a license to display the preprint in perpetuity. It is made available under aCC-BY 4.0 International license.

Supp Figure 1. Analysis of gRNA, Cas nucleases, and RNPs prior to subretinal injection in minipigs. **A.** Native gel electrophoresis of sgRNAs prior to eRNP assembly to evaluate RNA conformation**. B.** Native gel electrophoresis of assembled NHEJ-eRNPs and BE-eRNPs to determine quaternary structure. **C.** Denaturing PAGE and SDS-PAGE of NHEJ-eRNPs and BE-eRNPs to analyze purity of total RNA and protein. **D.** Dynamic light scattering of NHEJ-eRNP and BE-eRNP. gRNA annotations are described in Supp Table 2.

Supp Figure 2. Dynamic light scattering data for all test articles. **A.** BE-eRNP (assembled with sgRNA targeting Abca4 locus) at 3 different dosing concentrations. **B.** BE-eRNP (assembled with sgRNA targeting the Rosa26 locus) at 2 different dosing concentrations. **C.** NHEJ-eRNP (assembled with sgRNA targeting the Rosa26 locus) at a single dosing concentration.

Supp Figure 3. Enzymatic activity of NHEJ-eRNPs and BE-eRNPs. In vitro DNA cleavage (NHEJ-eRNPs) and adenosine deamination (BE-eRNPs) assay data are displayed.

Supp Figure 4. Identification of high efficiency NHEJ gRNAs targeting the mouse *Rosa26* locus and site-wise comparison of adenine base editing rates versus indel rates. **A.** Mouse T cell editing rates versus gRNA for NHEJ-eRNPs. NHEJ-eRNPs were complexed with various gRNAs targeting the *Rosa26* locus and then used to treat stimulated mouse T cells. NHEJ-eRNPs were complexed with the indicated gRNAs in crRNA:trcrRNA format and then nucleofected into cells.

bioRxiv preprint doi: https://doi.org/10.1101/2024.12.30.630799; this version posted December 31, 2024. The copyright holder for this preprint (which was not certified by peer review) is the author/funder, who has granted bioRxiv a license to display the preprint in perpetuity. It is made available under aCC-BY 4.0 International license.

Editing rates were quantified based on the frequency of reads containing indels from Illumina sequencing. The asterisk indicates the crRNA corresponding to the targeting gRNA used in main text Fig. 2B and 2C. **B.** Frequency of A→G transitions upon ABE-eRNP treatment vs. frequency of indels upon NHEJ-ABE treatment at each A position across protospacers tested in Fig. 2A and panel A of this figure. For the A position indicated in each panel, the ABE and indel rates for subset of gRNAs with an A at that position were plotted against each other. Each point corresponds to the mean editing rate for one gRNA and error bars represent the standard deviation across biological replicates.

Supp Figure 5. A. Representative images of murine eyes treated with subretinal administration of 2 µl of vehicle or eRNP at denoted time points with an untreated eye control. Sections were stained by anti-Cas9 immunohistochemistry (teal). **B.** Representative images of murine eyes 1 hour post subretinal administration with 1 µl eRNP stained with isotype control (left) or anti-Cas9 immunohistochemistry (right, teal) **C.** Editing rates by tissue following subretinal injection of *Rosa26*-targeting ABE-eRNPs complexed with mmRosa_sg1 in mice at four doses. Five days following subretinal administration, mice were sacrificed and their eyes dissected to separate the neural retina from the eyecup, and then RPE layer was separated from the choroid. Editing rates from Illumina DNA amplicon sequencing are shown as mean ± SD across eyes at each dose for retina and RPE tissue samples. Each overlaid point corresponds to a single eye. Reads were scored as positive for editing if at least one A→G transition was detected within a 10 base edit window. These data were collected with the same sample shown in Fig. 3 at a different study site and with a different operator.

Supp Figure 6. In vitro editing with *S. scrofa*-specific gRNAs and safety-related parameters following eRNP subretinal administration in minipigs. **A.** Adenine base editing and indel formation following nucleofection of BE-eRNPs (left panel) and NHEJ-eRNPs (right panel)

bioRxiv preprint doi: https://doi.org/10.1101/2024.12.30.630799; this version posted December 31, 2024. The copyright holder for this preprint (which was not certified by peer review) is the author/funder, who has granted bioRxiv a license to display the preprint in perpetuity. It is made available under aCC-BY 4.0 International license.

in porcine cell lines PK(15) and PT-K75 at various concentrations with two different gRNAs. Editing rates calculated from Illumina DNA amplicon sequencing are plotted as mean ± standard deviation from three biological replicates. The indicated RNP concentrations correspond to the final mixture with cells in the nucleofection cuvette. **B.** Editing rates as shown in (A) and (B) for eyes treated with a pool of ABE-eRNPs each complexed with a different gRNA. Editing rates corresponding to the intended gene target sequence are shown for each gRNA and arranged by tissue. **C.** Aggregate SPOTS scores per minipig porcine eye following subretinal administration of eRNP. **D.** Outer nuclear layer thickness measured by OCT plotted for NHEJ-eRNP groups. Data are plotted as the mean ± standard deviation for each group across all measurements in all eyes. **E.** Outer nuclear layer thickness measured by OCT plotted by eye and grouped by eRNP, gRNA, and dose. Data are plotted as the mean ± SD across measurement areas (regularly spaced 2D OCT slices spanning the superior and inferior regions proximal to the superior injection bleb) collected for each eye. **F.** Representative images of porcine eyes 2 weeks post subretinal administration stained with isotype control or anti-Cas9 immunohistochemistry (teal). Representative whole eye scans and high magnification (inset) images of H&E-stained porcine eyes 2 weeks post subretinal administration with **G**) 3 nmol or **H**) 3 nmol NHEJ eRNP.

Supp Figure 7. Adenine base editing in cDNA and indel formation following NHEJ-eRNP or BE-eRNP subretinal administration in minipig eyes. **A.** Editing rates by tissue following subretinal injection of *ABCA4*-targeting ABE-eRNPs complexed with sgABCA4 in minipigs. Two weeks following subretinal administration, animals were sacrificed and eyes were dissected to collect intact tissue layers for the neural retina and the choroid + retinal pigmented epithelium. For each tissue, three biopsy punches were taken corresponding to the injection bleb, tissue immediately adjacent to the bleb, and a region distal to the bleb. Editing rates observed in reverse-transcribed cDNA from each sample from Illumina amplicon sequencing are plotted for each eye by tissue and dose as described in Fig. 4. **B.** Editing rates by tissue following subretinal injection

bioRxiv preprint doi: https://doi.org/10.1101/2024.12.30.630799; this version posted December 31, 2024. The copyright holder for this preprint (which was not certified by peer review) is the author/funder, who has granted bioRxiv a license to display the preprint in perpetuity. It is made available under aCC-BY 4.0 International license.

of *ROSA26*-targeting NHEJ-eRNPs complexed with sgRosa26 in minipigs at multiple doses. Editing rates from Illumina DNA amplicon sequencing are plotted for each eye by tissue, biopsy region, and dose. Reads were scored as positive if they had an InDel at the expected cut site.

Supp Figure 8. SRM1 eRNP test article characterization and evaluation of activity in SRM1 pigs following subretinal eRNP administration. **A.** Plasmid cutting by purified NHEJ-eRNP samples complexed with non-targeting (NT) and reporter locus gRNAs (SRM1). Sample aliquots were collected and stored before shipment to the test site (pre-injection) and from the material recovered from the injector following administration (post-injection), and then assayed side-by-side. Each point corresponds to a separate reaction. **B.** Outer nuclear layer thickness according to OCT at different time points post injection following NHEJ-eRNP administration in the SRM1 pig study described in Fig. 5. Data are plotted as the mean ± standard deviation for each group across all measurement areas from superior (injected) regions. **C.** Aggregate SPOTS scores per porcine eye following subretinal administration of eRNP. **D.** Fluorescent images of a wild-type littermate (from SRM1 strain) porcine eyes treated with either vehicle or targeting gRNA NHEJ-eRNP. **E.** Fluorescent images from a SRM1 porcine eye from either the uninvolved inferior region, stained without anti-tdTomato primary antibody, or with isotype antibody (corresponding to the anti-tdTomato antibody). **F.** Fluorescent image at 4× and 20× magnification within the treated (superior) and uninvolved (inferior) region of a SRM1 porcine eye. **G.** Fluorescent images of a flat-mounted RPE/Choroid/Sclera from a wild-type littermate control (from SRM1 colony) eye at 4× and 20× magnification.

Supplemental Figure 1

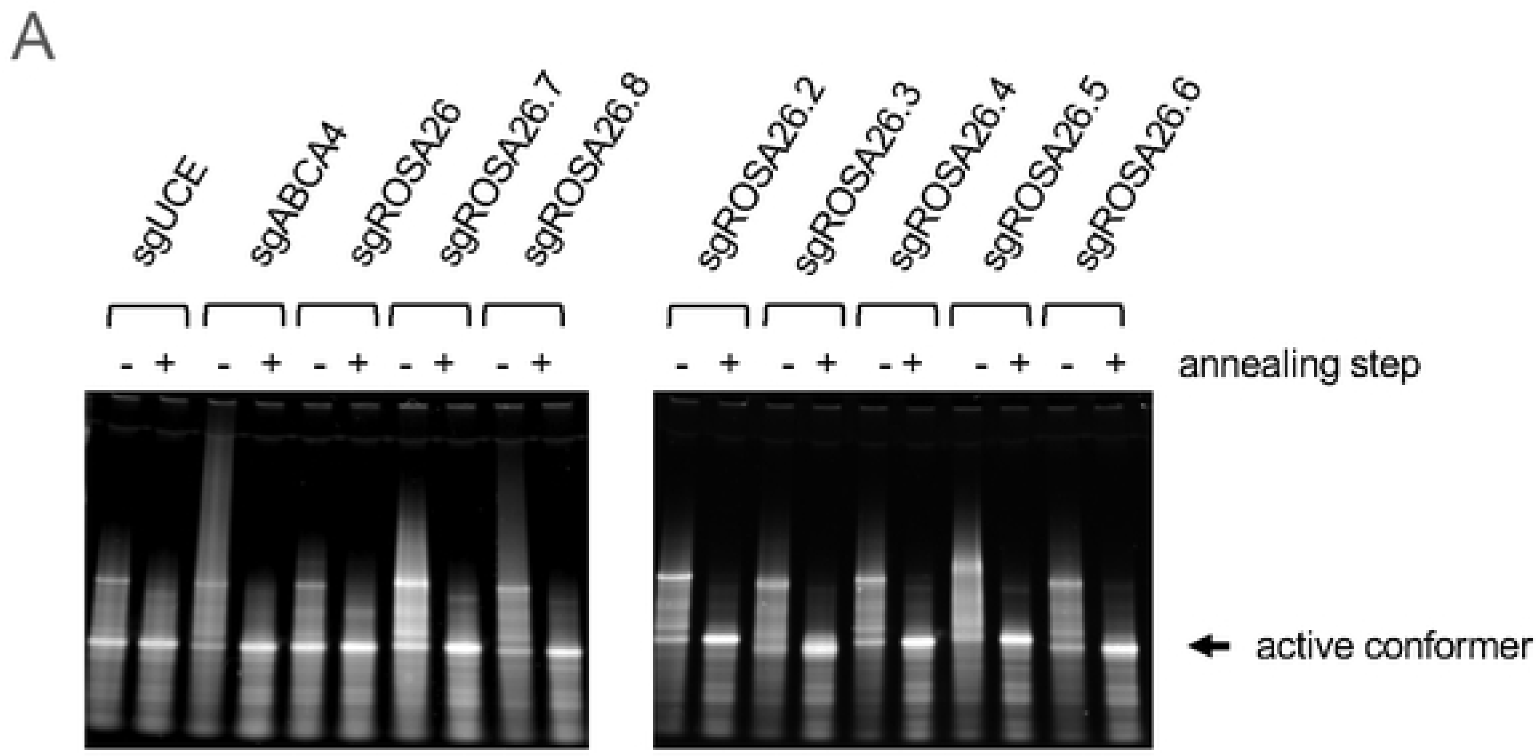

bioRxiv preprint doi: https://doi.org/10.1101/2024.12.30.630799; this version posted December 31, 2024. The copyright holder for this preprint (which was not certified by peer review) is the author/funder, who has granted bioRxiv a license to display the preprint in perpetuity. It is made available under aCC-BY 4.0 International license.

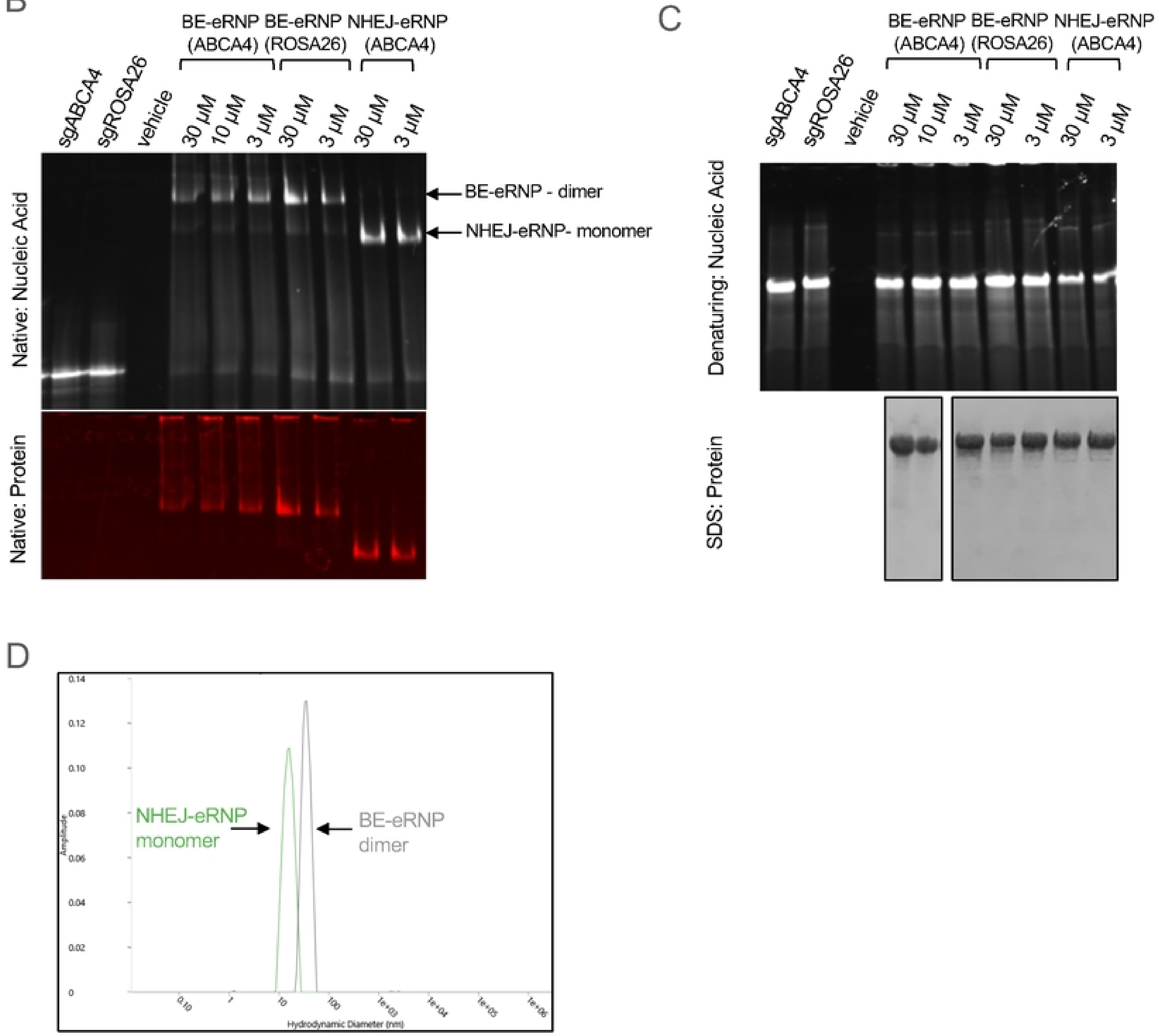

Supplemental Figure 2

A

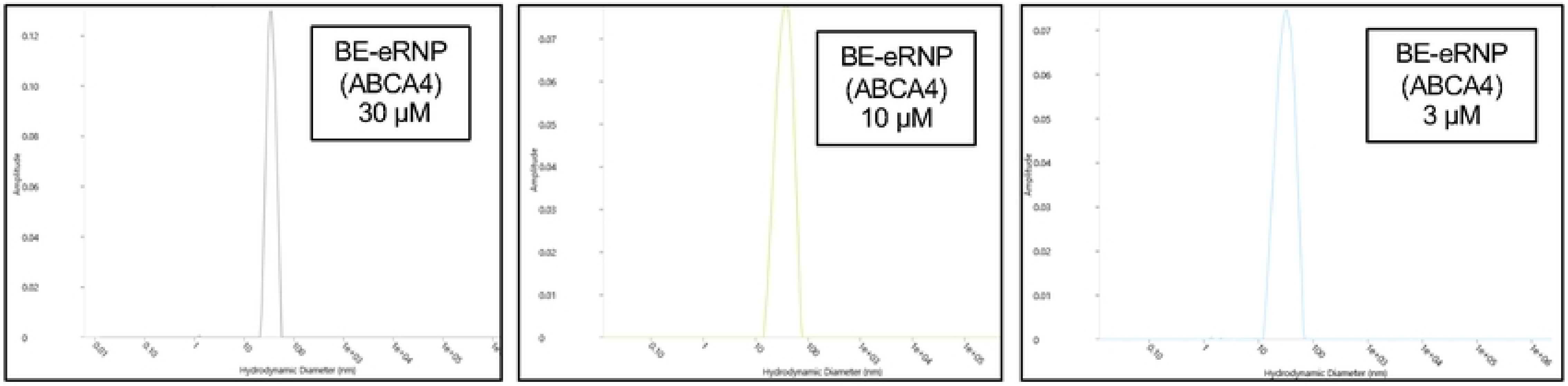

B

bioRxiv preprint doi: https://doi.org/10.1101/2024.12.30.630799; this version posted December 31, 2024. The copyright holder for this preprint (which was not certified by peer review) is the author/funder, who has granted bioRxiv a license to display the preprint in perpetuity. It is made available under aCC-BY 4.0 International license.

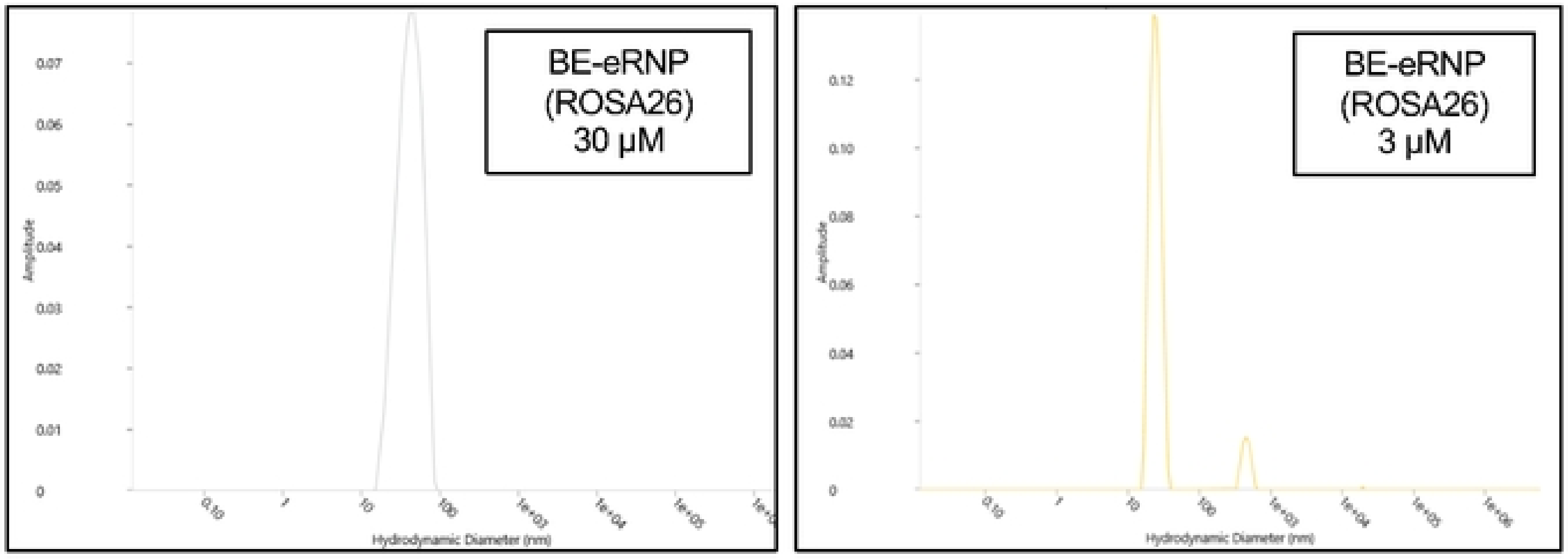

C

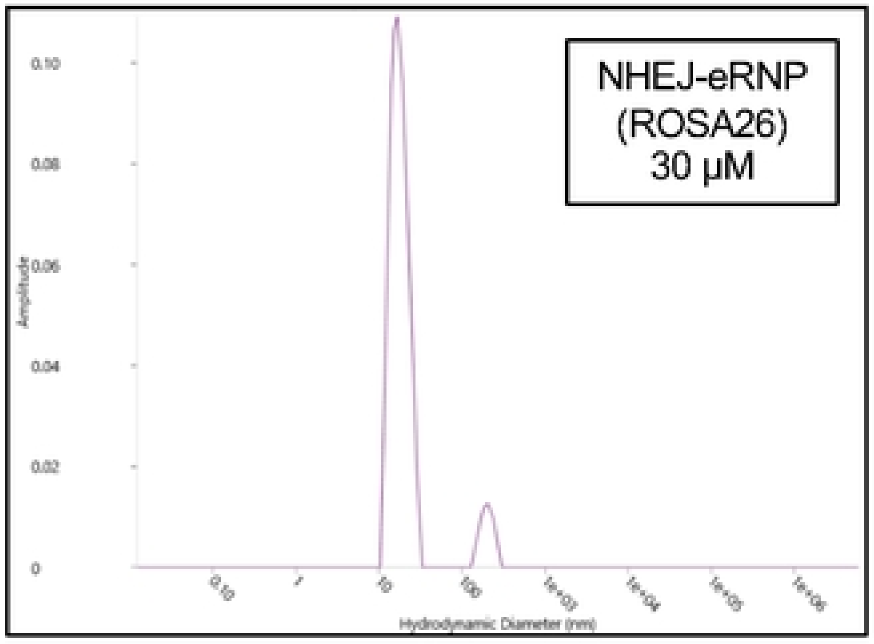

Supplemental Figure 3

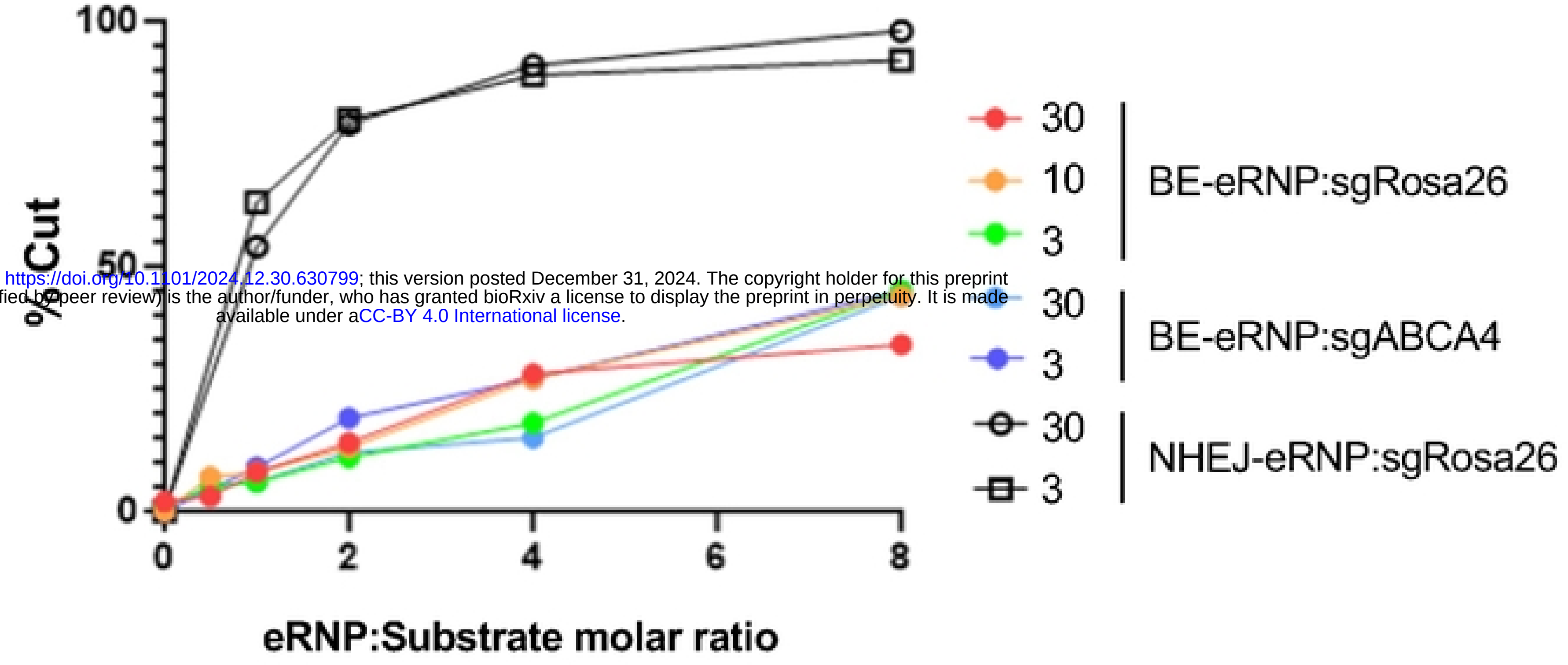

bioRxiv preprint doi: https://doi.org/10.1101/2024.12.30.630799; this version posted December 31, 2024. The copyright holder for this preprint (which was not certified by peer review) is the author/funder, who has granted bioRxiv a license to display the preprint in perpetuity. It is made available under aCC-BY 4.0 International license.

Supplemental Figure 4

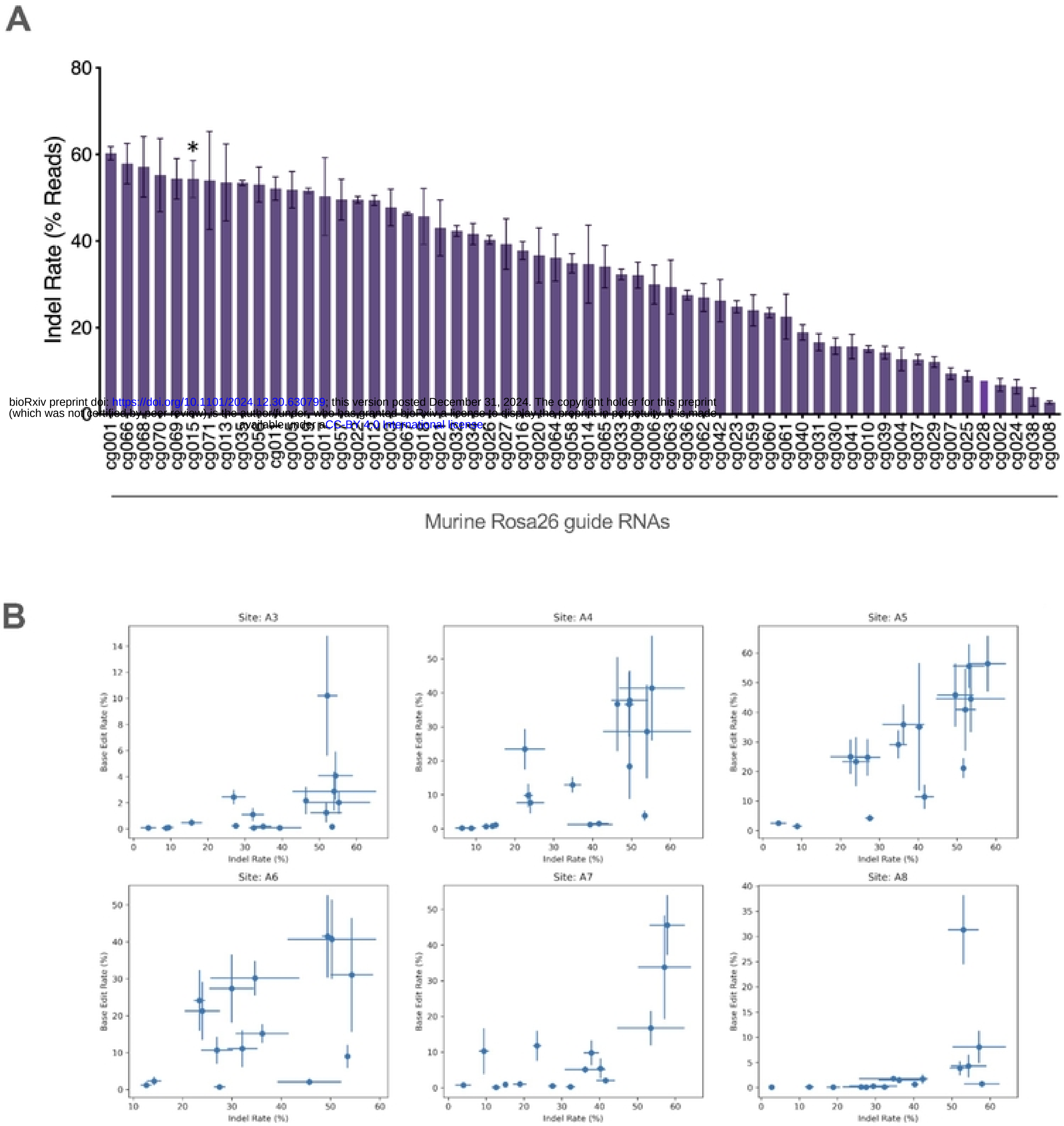

bioRxiv preprint doi: https://doi.org/10.1101/2024.12.30.630799; this version posted December 31, 2024. The copyright holder for this preprint (which was not certified by peer review) is the author/funder, who has granted bioRxiv a license to display the preprint in perpetuity. It is made available under aCC-BY 4.0 International license.

Supplemental Fig 5

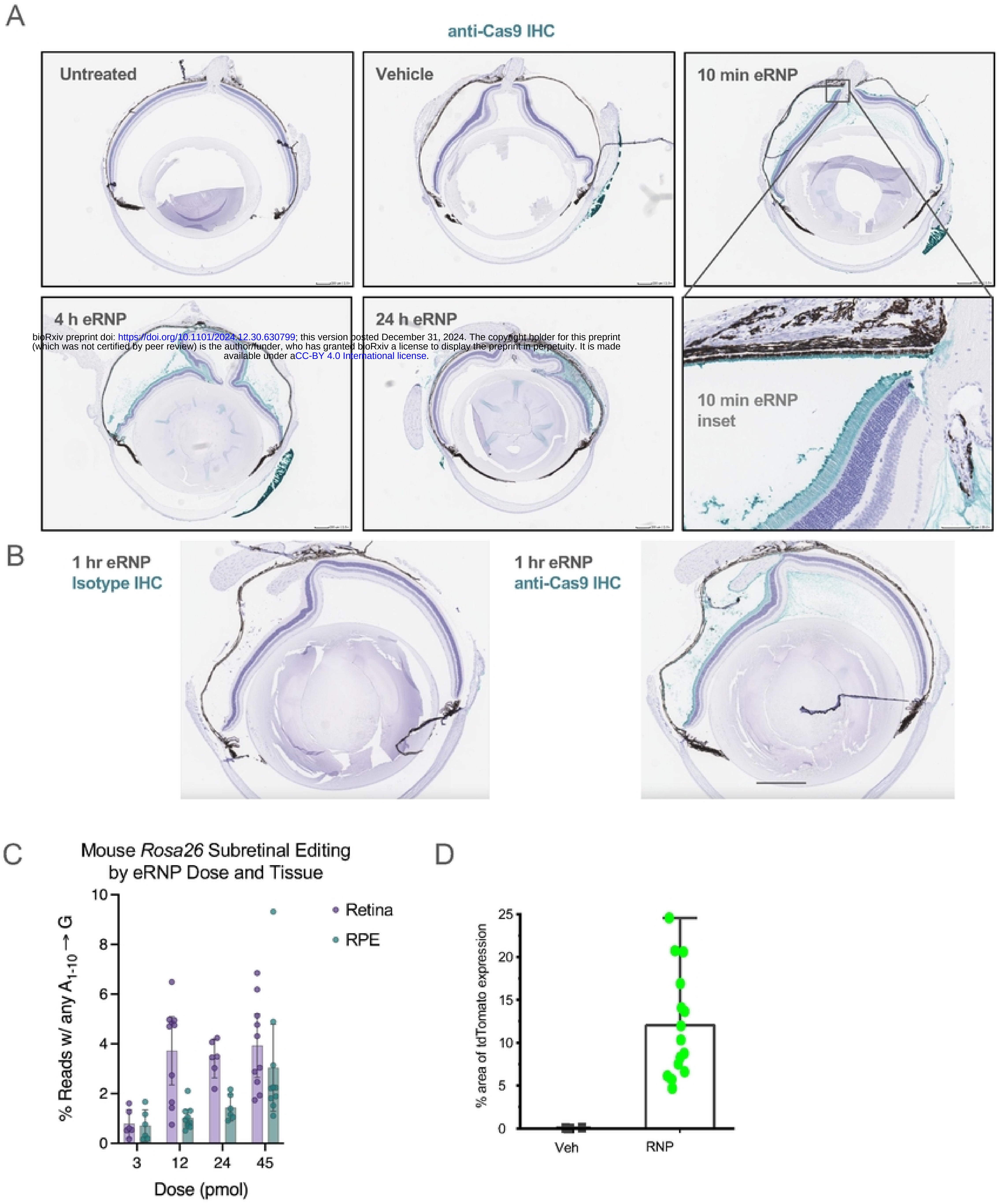

bioRxiv preprint doi: https://doi.org/10.1101/2024.12.30.630799; this version posted December 31, 2024. The copyright holder for this preprint (which was not certified by peer review) is the author/funder, who has granted bioRxiv a license to display the preprint in perpetuity. It is made available under aCC-BY 4.0 International license.

Supplemental Figure 6

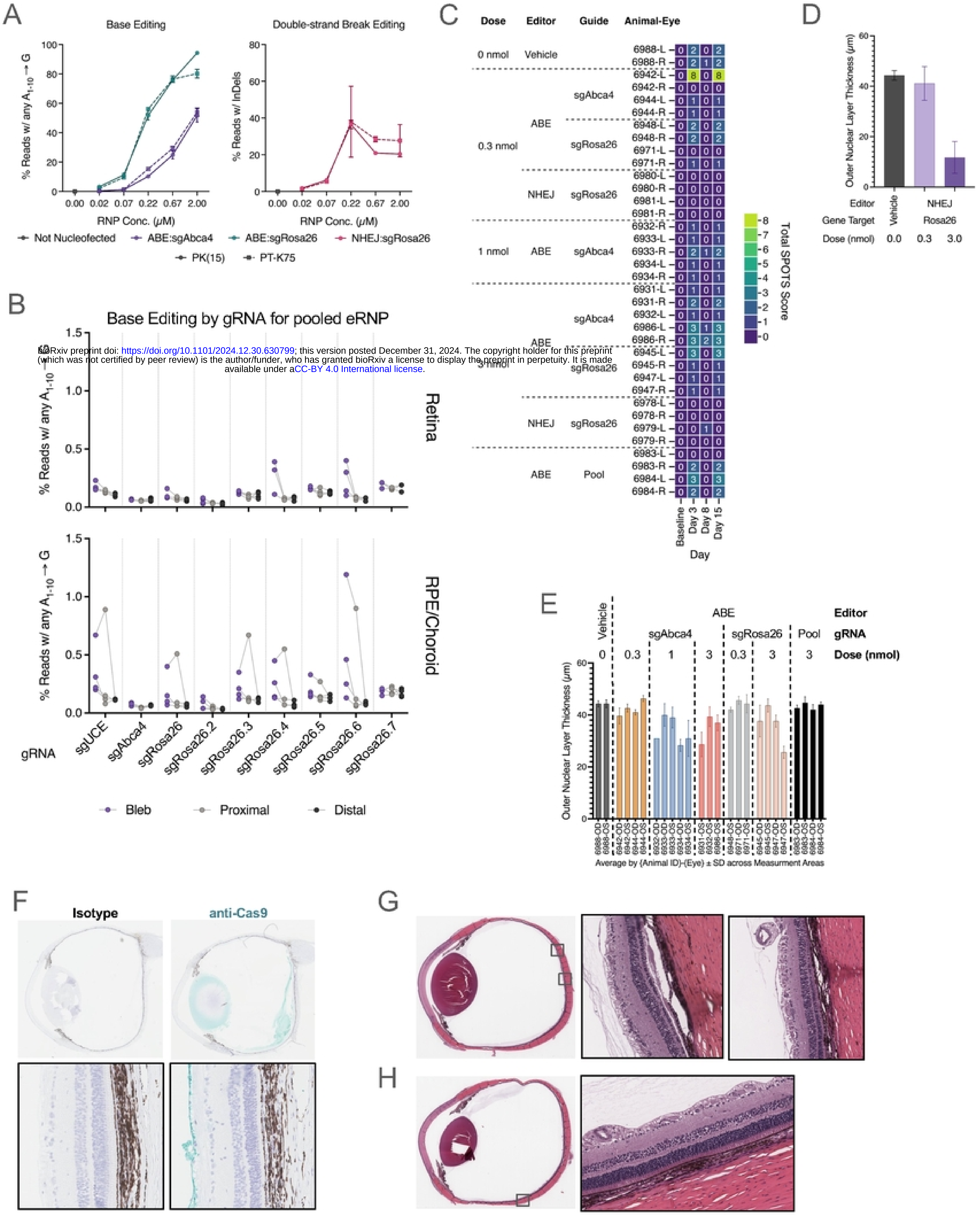

bioRxiv preprint doi: https://doi.org/10.1101/2024.12.30.630799; this version posted December 31, 2024. The copyright holder for this preprint (which was not certified by peer review) is the author/funder, who has granted bioRxiv a license to display the preprint in perpetuity. It is made available under aCC-BY 4.0 International license.

Supplemental Figure 7

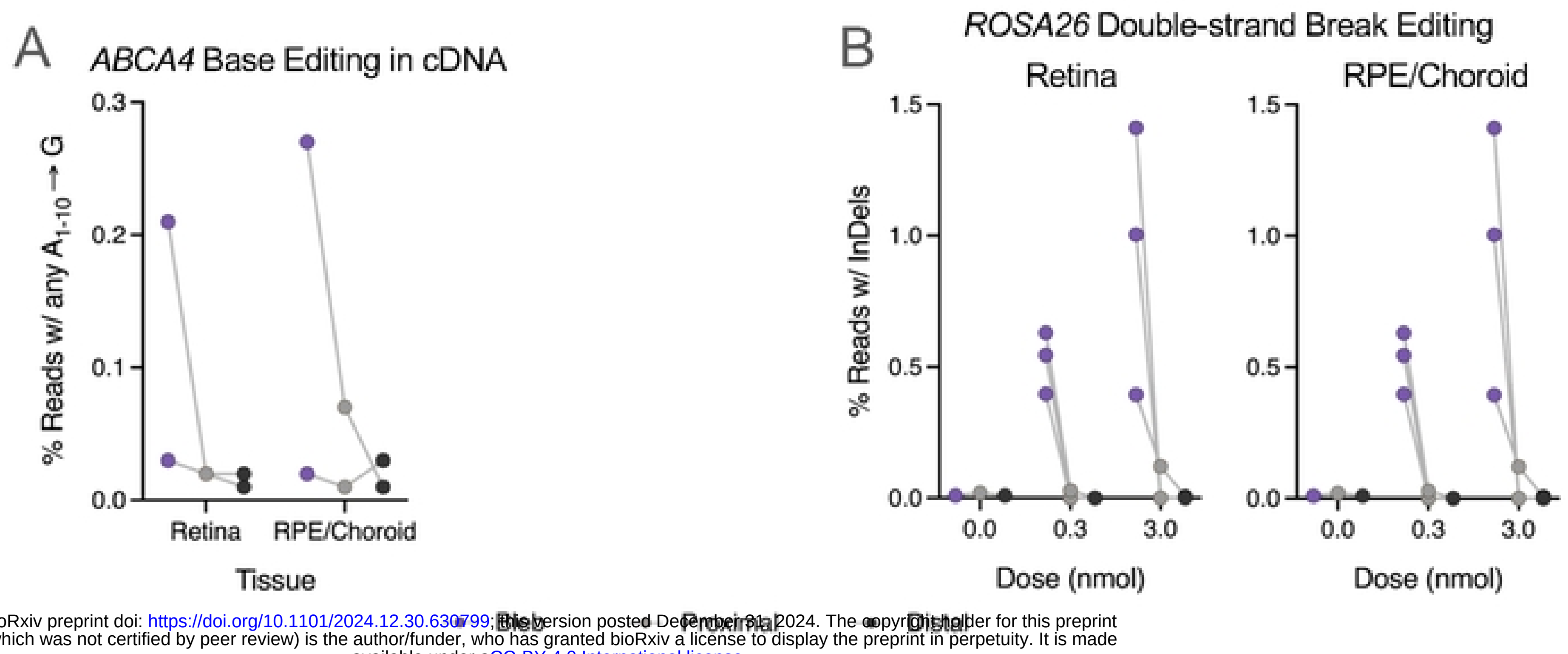

bioRxiv preprint doi: https://doi.org/10.1101/2024.12.30.630799; this version posted December 31, 2024. The copyright holder for this preprint (which was not certified by peer review) is the author/funder, who has granted bioRxiv a license to display the preprint in perpetuity. It is made available under aCC-BY 4.0 International license.

Supplemental Figure 8

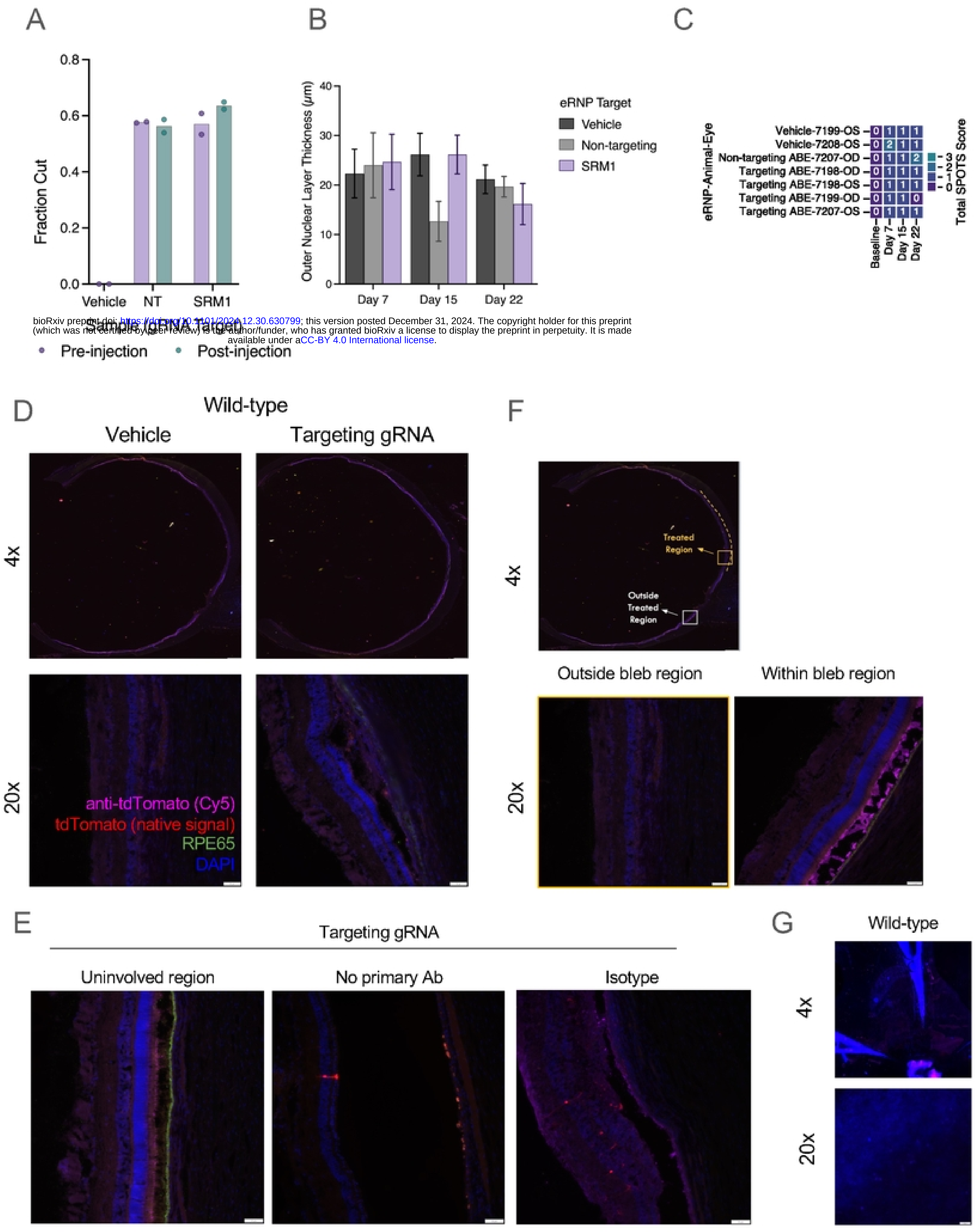

bioRxiv preprint doi: https://doi.org/10.1101/2024.12.30.630799; this version posted December 31, 2024. The copyright holder for this preprint (which was not certified by peer review) is the author/funder, who has granted bioRxiv a license to display the preprint in perpetuity. It is made available under aCC-BY 4.0 International license.
